# An Environmental Scan and Qualitative Inquiry of Cancer Patient Navigation Services in North Carolina

**DOI:** 10.21203/rs.3.rs-4189013/v1

**Published:** 2024-04-12

**Authors:** Leila Fathi, Karl Umble, Austin R. Waters, Erin E. Kent

**Affiliations:** North Carolina State University; University of North Carolina at Chapel Hill; University of North Carolina at Chapel Hill; University of North Carolina at Chapel Hill

## Abstract

**Background and Objectives:**

Patient navigation services reduce barriers to accessing cancer care and lead to improved outcomes for patients. North Carolina (NC) has thousands of cancer patients seeking cancer care services each year. We sought to complete a digital environmental scan and qualitative inquiry of cancer patient navigation services throughout the state to better inform patients, hospitals administrators, and state officials about the current state of patient navigation programs for cancer patients throughout NC.

**Methods:**

For seven cancer hospitals in NC, two steps were used: an environmental scan of publicly available information on the hospitals’ websites about navigation services, and key informant interviews with navigation staff at each site.

**Results:**

The website scans revealed information about navigation services was incomplete. Each hospital had a page dedicated to cancer navigation, but many did not outline the specific services available to patients. Interviews revealed that navigation services are available to cancer patients across diagnoses, although only a subset of patients receive services. Cancer navigators reported that their work includes care coordination, patient advocacy, emotional support, and addressing non-medical barriers to health care access (transportation, finances, childcare, etc.). Each navigation service had a unique configuration and referral pattern.

**Conclusions:**

Cancer hospitals throughout NC are working to address barriers to care commonly faced by patients, with some programs offering more robust services compared to others. Hospitals would benefit from updating their websites at regular intervals to fully report the services available to patients through their programs, including direct and clear patient navigation contact methods.

## Introduction

A cancer diagnosis is often accompanied by a number of complex care coordination issues. Cancer patients manage scheduling and attending appointments with multiple specialists, filling and taking on average 5.5 prescription medications, and facing significant time and financial burdens [1, 2]. In addition to the physical toll of cancer and treatment, patients and their caregivers must coordinate appointments, treatments and prescription management, often on their own. Patient navigation aims to help patients overcome such barriers to care and reduce disparities to promote access to care and good health outcomes [1].

Patient navigators guide patients through the healthcare system and coordinate a broad range of services such as screening, diagnosis, treatment, follow-up care, appointment scheduling, and communication with healthcare providers [2]. Not only do patient navigators work to coordinate care and overcome barriers to care, but they also work to overcome disparities. If operationalized appropriately, patient navigation can be used as an equity-based intervention as it works to close gaps in access [3]. Navigators may also be considered to be patient advocates, given their role in helping patients and their families communicate with insurance companies, employers, and lawyers in order to ensure the patient receives necessary services [2]. Evidence indicates that “patient navigation improves quality of life and patient satisfaction with care in the survivorship phase and reduces hospital readmission in the active treatment and survivorship care phases [1].” Additionally, emerging literature also suggests that patient navigation can result in higher cancer screening, treatment adherence and follow-up rates, higher patient satisfaction, and earlier tumor detection [4]. Given the complex systems and barriers that patients and their families may face, patient navigation can be an effective way to assist patients and families in receiving access to needed services to reduce the burden of navigating complex US health systems [5].

Various models of patient navigation include different roles; for example, some programs utilize nurse navigators while others employ lay navigators (professional, non-clinical) or volunteer navigators [5]. Regardless of the type of navigator used, a core component of these programs are barrier assessments. These barrier assessments are conducted by navigators alongside the patient to identify barriers the patient may face in receiving their care. There is not a standard barrier assessment tool utilized by oncology patient navigation programs, however, many of the questions center around The Supportive Care Framework for Cancer Care [6]. This framework was developed as a tool to help cancer care professionals understand what type of assistance cancer patients may need [7]. Much of the work of navigation centers around the 7 domains of patients’ needs identified by the framework: physical, informational, emotional, psychological, social, spiritual, and practical. This conceptual framework is the basis for many cancer patient navigation programs, but each program has a unique method of navigating patients and little literature exists surrounding cancer patient navigation program commonalities in features and services within North Carolina (NC).

In 2022, over 65,000 new patients sought care at NC’s 11 hospital systems that provide cancer care [8, 9]. The types of navigation services available to these patients throughout the state have not been consolidated nor reported. Thus, we conducted a multi-method environmental scan of cancer patient navigation in NC to describe the state of cancer patient navigation, highlight common features, and identify gaps.

## Study Design and Methods

Environmental scans are a common approach to evaluate health services from the consumer perspective [10]. Environmental scans often include passive (e.g., assessing available information) and active (e.g., gathering information to assess) approaches to evaluation, both of which were conducted in this study [10]. The research team first searched and evaluated public-facing websites of NC cancer hospitals. Then conducted structured interviews with patient navigation program leaders at NC cancer hospitals to gain a more comprehensive understanding of the available services [11].

### Data Collection

First, we conducted internet searches on cancer hospital patient navigation services to determine the following: ease of identification and the extent of available patient navigation information (Table 1). We used the health system’s website to navigate to their respective cancer patient navigation webpages from the patient perspective. If we identified a specific cancer navigation page on their website, we evaluated the elements describe in Appendix A for each navigation webpage.

The patient-facing websites of each hospital were then used to identify points of contact for individuals involved in cancer patient navigation program. The study team recruited contacts via phone and email. Out of the eleven systems that we approached to participate in the study, seven (64%) responded and agreed to participate and scheduled a Zoom interview.

One structured interview was conducted by LF per site. Participants were either a patient navigator or a navigation program director. The interview guide was developed and refined by the undergraduate researcher and public health researchers with experience in evaluation of public health programming and cancer health services research (Appendix A). Questions were aimed to get an understanding of the current and future state of each hospital’s cancer patient navigation program, including structure and desired future direction. For consistency, informants from the hospital systems were asked the same set of questions in the same order. Participant respondents were recorded via detailed written notes, and video/audio was recorded for reference.

### Qualitative Analysis

The overarching approach for this study was a deductive content analysis [12]. Publicly available online data were collected and consolidated into Table 1. Following each structured interview, the researchers consolidated notes taken during the interview, supplemented by information from the audio recordings, into a data summary for each hospital. Each interview participant was then asked to check the accuracy of the information collected and chosen to be displayed in the study [13]. All participant edits were incorporated in Tables 1–4. The study team then analyzed the information collected from both sources for patterns and commonalities. This study was led by a student at the UNC Gillings School of Public Health (LF) as part of their honor thesis and was guided by 2 faculty members (KU, EEK) and a doctoral student (ARW). The COREQ checklist reporting qualitative research was used [14].

The Institutional Review Board at the University of North Carolina at Chapel Hill reviewed this study, IRB# 22–3005, and deemed it Non-Human Subject Research. All participants gave their full consent before, during, and after the interviews.

## Results

As reported in the structured interviews with 7 health systems, size of the health system encompassing the cancer hospital ranged from 3,000 to 70,000 employees. Patient navigation programs began as early as 2004, with the latest program being introduced in 2021. All the hospitals studied report having navigation services available to all cancer patients. Type of navigator varied across sites with the most common type being nurse navigators (n=7), followed by lay navigators (n=3) and volunteer navigators (n=2).

### Description of Publicly Available Online Patient Navigation Information (Table 1)

Nearly all health systems had dedicated pages on their website for cancer patient navigation (n=6). However, the one health system that did not have a separate cancer patient navigation section of their website did provide a number for patients to call. Specific navigation services available to patients were not outlined on the websites of 2 out of the 7 hospitals. However, the 3 out of the 4 hospitals that outlined the navigation services available on their website included services such as patient education, scheduling, assistance with managing side effects and symptoms; the other 1 out of 4 hospitals provided a more general description of services such as coordination of care. In terms of providing contact methods for patients on their website. The hospitals had a mix of contact methods: patient navigation online inquiry form, email address, phone number to general cancer support services, phone number for the broader general medical center, phone number for scheduling cancer care appointments, as well as direct phone numbers for navigators. A patient navigation online inquiry form was used at 1 hospital, 1 hospital used an email for general support services, 1 hospital had no contact method, 2 hospitals had a phone number for general cancer support services, and 1 hospital listed a phone number for a featured nurse navigator as well as the general phone number for the hospital.

### Qualitative Description of Patient Navigation Programs & Services Provided (Table 2)

Hospitals utilized a combination of disease-specific navigators, as well as non-disease specific navigators. Navigators at all hospitals played a key role in coordination of care and patient education. Some of the most common services that navigators connected patients to included financial assistance, social work, emotional support/counseling, dietician services, palliative care, genetic counseling, fertility preservation, hospitality homes, rehabilitation services, and transportation assistance. Some of the hospitals had longer resource lists compared to others. The overall depth of resources seemed to vary by hospital due to location and local resources. For example, a hospital in the more rural part of the state did not have the ability to connect patients to the same number of resources compared to a hospital in a more metropolitan region of the state. Some of these extra resources in the more metropolitan part of the state include things like wellness programs such as exercise, yoga, and nutrition services, as well as robust rehabilitation programs and support groups for both the patient and family members. In addition to connecting patients with these services, many of the hospitals within the study had a cancer center where patients could use computers, seek food/water, as well as other amenities. One hospital even had massage chairs and a resource library for patients to utilize.

#### Types of Navigators at Each Health System

The three main types of patient navigators available to patients at the hospitals studied include clinically trained nurse navigators, lay navigators, and volunteer navigators. 2 out of the 7 hospitals studied provide navigation services through all three types of navigators: clinically trained nurses, lay navigators, and volunteer navigators. 1 out of the 7 hospitals studied provide services through clinically trained nurse navigators and lay navigators, without the use of volunteer navigators. The rest of the hospitals studies (4 out of 7) provide navigation services solely through clinically trained nurse navigators.

#### Configuration and Referral Patterns of Patient Navigation Services at Each Health System (Table 3)

##### Referral:

Generally, patients are identified within the hospitals system and are contacted to conduct a barrier assessment. At 1 of the 7 hospitals, Hospital G, patients may self-refer for navigation services.

Connecting with the Patient/Diagnosis: Generally, navigators make contact with patients around the point of diagnosis. At 5 out of the 7 hospitals, navigators intend to meet with patients prior to their first appointment to connect and conduct a barrier assessment. While at 1 out of the 7 hospitals, the navigator meets with patients at their first oncology visit to establish care. At all 7 hospitals, an emphasis is placed on the beginning of the patient’s journey, starting at diagnosis and 1^st^ consultation. A barrier assessment is completed around the time of initial contact with the patient at all 7 hospitals. At all 7 hospitals, patients are contacted either right before or after their first appointment. A barrier assessment is completed around this time as well. However, at hospital G, for breast, lung, and GI patients, navigators may intervene at the point of biopsy.

##### Treatment:

At all 7 hospitals, patients are followed through their course of treatment. At one of the hospitals specifically, volunteer navigators exclusively follow up with patients in person while an oncology nurse navigator follows up with patients via phone. At one of the 7 hospitals, hospital D, a social work navigator conducts a barrier assessment at multiple points throughout the patient’s treatment continuum, including after the first appointment, after the start of treatment and once treatment has finished. 3 out of the 7 hospitals noted that navigators meet with patients in person as much as possible. The other 4 hospitals did not specify whether patients are followed up with patients in person or through phone.

##### Survivorship:

At one of the 7 hospitals, hospital D, a final barrier assessment is completed during the first survivorship visit. Only one of the 7 hospitals, hospital D has a survivorship program. This program is run by social work navigators, not very robust.

### Future Direction of Patient Navigation for Cancer Patients in North Carolina (Table 4)

#### Future direction at each health system:

Overall, all hospitals wish to bolster their programs by increasing navigators and the number of available support services to ensure that every patient has access to navigation services if needed and desired. Specifically, 4 out of the 7 hospitals mentioned increasing the number of navigators across disease groups within their programs in order to bolster services and cover all disease groups. One hospital noted that they wish to switch to disease-specific navigators at their satellite facilities as opposed to general navigators. Additionally, 3 out of the 7 hospitals noted that they wish to improve their forms, data collection, and tracking. Two hospitals noted that they wished to do this in order to demonstrate the positive downstream financial impacts of patients; the other hoped it would better inform their process. 2 out of the 7 hospitals mentioned improving their standard of work, including how they triage patients based on need and how they follow patients throughout their care.

#### Desired future direction of patient navigation in general:

Hospitals wish to see an expansion of patient navigation into other chronic diseases. Hospitals also hope that navigation becomes more financially sustainable. While one hospital noted that they wished for this service to be made reimbursable, others were intent that these services do not become a billable service so as not to risk these services becoming at cost to the patient. They want coverage for navigation services by payers without cost to the patient, as the patients who need these services the most are often financially at-risk patients. They wish to have support across health care sectors for navigation and greater accessibility to these programs for patients while also increasing the financial sustainability for these programs for health systems.

## Discussion

As evidenced by the findings of this study, cancer centers throughout North Carolina offer robust patient navigation services for patients with some programs offering more services compared to others. These hospitals report that they make their patient navigation services available to all cancer patients, and that their services range from coordination of care to connection with community resources. While the patient navigation programs studied were robust, review of their websites revealed that their digital presence generally did not reflect their full range of services.

When comparing results from our digital environmental scan and qualitative interviews we found that a majority of information about patient navigation programs is missing from hospital webpages. This finding suggests that hospitals may not be utilizing their websites to convey the full extent of their programs. For example, some of the hospitals offered more services to patients than what was described on their websites. In the context that a vast majority of adults in the United States (81%) being digitally literate, updating hospital websites to display the full extent of their services may result in increased use of PN services and thus reduced barriers to care [15]. The necessity of digital information is particularly important as the US population ages and those born into a society with technology-based communication enter into age groups where cancer is most commonly diagnosed [16].

Additionally, methods for contacting patient navigation support services are mixed, with some hospitals providing clear and direct contact methods for patient navigation services while others provided no contact method at all or one that leads patients to general cancer care services. As a result, patients seeking information about patient navigation services may not understand the full extent of services offered at the hospitals they are researching, and they may be left feeling confused without clear and direct contact methods for patient navigation services at their desired hospital. Hospitals may have opportunities for improvement in this regard, as prospective patients looking for specific patient navigation services may choose to seek care elsewhere if the information is not readily accessible on the hospital’s webpage and if the contact method is vague or cumbersome. While many patients have little choice over where they will receive care, some may have the privilege to choose. If direct contact information is not easily available, it leads to the potential of patients not being connected with a navigator at all. Patient navigation improves quality of life and reduces hospital readmission [1]. Additionally, administrative tasks can lead to patients and their caregivers delaying or forgoing care [17]. Having more information readily available on hospital websites may reduce patient and caregiver administrative burden and potential lead to less patients delaying or forgoing care. Given this, having direct and clear contact information to connect patients with these essential service has the potential to improve outcomes for patients.

While all seven hospitals studied reported that they make their services available to all patients none reported systematic approaches for patient identification, risk stratification, and navigation. Maximizing the accessibility of patient navigation for those most in need should be prioritized. Pragmatically, not all patients need navigation; however, recent literature that revealed that less than a quarter of cancer patients at a university health system received patient navigation, suggesting that many patients in need are not being served [18]. Patient navigation programs in NC may benefit from taking an equity-based approach to their services to ensure barriers to care are mitigated via navigation. This may be particularly true in the context of the expansion of patient navigation, including programs that offer financial navigation [19, 20]. Future research should focus on understanding who receives which patient navigation services and how patient navigation programming can be effectively targeted.

### Limitations

We did not have the opportunity to meet with a key informant at every health system in NC that provides cancer care; thus, there may be cancer patient navigation programs that are not represented in this study. However, we were able to collect rigorous, participant-checked qualitative data and an assessment of publicly available digital information for most cancer hospitals that treat cancer patients in NC. Additionally, this study was cross sectional and specific to NC, thus limiting its generalizability. However, NC has an increasingly aging population making it a prime state to study PN services [21]. The findings of this study may be useful for other states with similar geographic and demographic patterns as NC. Additionally, our findings are directly applicable to cancer patient navigation programs throughout NC as they strive to provide robust patient navigation services.

## Conclusion

Hospitals that provide cancer care throughout NC health systems are working beyond the call of medicine to address the non-medical barriers to cancer care that many patients face. In doing so, patients who otherwise would not have been able to access their cancer treatments are now able to get to the hospital through transportation and childcare assistance, among a number of other resources, coordinated by the patient navigation team alongside social workers. Cancer patient navigation programs throughout NC include a broad range of services from coordination of care to connection with community resources. While some programs are more robust compared to others, this work is critical for thousands of North Carolinians. Finally, hospitals would benefit from ensuring that their digital patient-facing information accurately reflects their programs including contact information to maximize access to their patient navigation services.

## Figures and Tables

**Table 1.** North Carolina Hospital Patient Navigation Characteristics and Publicly Available Online Patient Navigation Information in 2023

	North Carolina Cancer Hospitals						
	A	B	C	D	E	F	G
**Hospital PN Characteristics**							
Size (number of employees)	33,000	70,000	3,000	12,000	29,000	11,000	8,000
PN program introduced (year)	2014	2009	2009	-	-	2004	-
Full PN program began (year)	2021	2012	2014	2014	2005	2006	2012
PN available to all patients	X	X	X	X	X	X	X

Type of PN							
Nurse navigator	X	X	X	X	X	X	X
Lay navigator	X		X	X			
Volunteer navigator	X			X			

**Online PN Information**							
Number of clicks from hospital home page	3	4	4	-	3	4	5

Information on type of services	Y	Y	N	N	N	Y	Y
Education		X				X	
Support		X					
Resources	X					X	
Care coordination/referrals	X	X				X	X
Scheduling							
Financial assistance		X					
Access issues		X					
Symptom management						X	
Type of PN	Y	N	N	N	N	N	N
Nurse navigator	X						
Lay navigator	X						
Volunteer navigator	X						

PN contact method	Y	Y	N	Y	Y	Y	Y
Online inquiry form	X						
Email	X	X					
Phone (non-PN specific number)				X	X	X	X
Phone (PN specific number)							X

Footnote: Y/N indicate if information was publicly available online.

X’s indicate the presence of each characteristic from the study participants perspective or publicly available online information.

**Table 2.** Description of Cancer Patient Navigation Programs Among North Carolina Cancer Hospitals, 2023

Cancer Hospital	Cancer Patient Navigation Program Description	Cancer Patient Navigation Services Provided
	Main Hospital:	OPNs address the non-medical barriers to care by connecting patients with the following services:
A	Three-tiered navigation program comprising of Oncology Nurse Navigators (ONN), Oncology Patient Navigators (OPN) and Oncology Volunteer Navigators (OVN) working collaboratively to improve patient outcomes and provide support ONNs-clinically trained oncology nurses embedded within a clinical care team managing and coordinating the patient’s cancer care OPNs-non-licensed full-time staff who are experts in non-medical barrier resourcing OVNs-trained to provide emotional support during treatment as well as conduct barrier assessments	Financial, psychological, and logistical supports Referrals to Interdisciplinary Services (Social Work, Financial Navigation, Integrative Medicine, Exercise Programs, Dietitians, Legal Assistance etc.) Emotional Support & Counseling Services Transportation, Lodging and Housing Caregiver services and support groups
		Care coordination is the responsibility of the clinically based ONN
		OVNs provide emotional support in person and over the phone. They report relevant information back to the navigation team who can work to address new concerns
	Main Hospital:	Patient navigators assist in coordination of care services such as scheduling as well as prescription assistance. Additionally, the navigators connect patients to the following resources:
B	Tumor site-specific navigators cover all malignant tumor sites	
	Satellite Facilities: General navigators cover all tumor sites	Cancer resource center with access to computers/internet Transportation/Gas cards Financial assistance Counseling Rehabilitation services Therapists
	Main Hospital:	Navigators assist in coordination of care, provide patient/caregiver education, advocate for patients, and conduct barrier assessments/resolution. Additionally, the navigators connect patients to the following resources:
C	3 types of navigators-nurse navigator, patient navigator and resource navigator Nurse navigators follow the patients with the highest acuity; patients are followed through the treatment phase. Patient Navigators follow special populations of patients who may be vulnerable, at risk for falling through the cracks, or those with a higher no-show rate, or have compliance concerns Resource navigators assist patients with concreate barriers for example: transportation, housings, childcare. This type of navigator works alongside the nurse navigator and patient navigator to assist the patient, or can work alone. Once the barrier is eliminated the patient is discharged from resource navigation services.	Psychological support/counseling Financial resources Dieticians Exercise therapy Support groups Transportation resources/assistance Other community resources
	Satellite Facility:	
	All navigators are clinically trained nurses and cover all disease groups. The nurse navigators reach out to all cancer patients and follow those who need assistance. Patients are met or contacted at the first visit and followed through treatment. The nurse navigators provide services from patient education to care coordination.	
	At both Cancer Centers, oncologist, nurses, other health professionals, family members and the community can make a navigation referral.	
	Main Hospital:	
D	Nurse navigators follow the patient throughout the treatment progression Social work navigators follow up on social needs and connect to resources Lay navigators assist nurse navigators and social work navigators Distress screenings are completed at 4 pivotal points throughout the patients’ care: initial diagnosis, radiation treatment start, radiation treatment completion, 1^st^ survivorship visit	Disease site-specific nurse navigators meet with patients at initial diagnosis, coordinate between physicians, provide patient education, make referrals, follow patient through treatment continuum, and work closely with social workers Social work navigators complete initial distress screenings with patients after 1^st^ appointment and follow up on resource needs identified. Additionally, social workers run support/survivorship programs Lay navigators (if available) assist with updating community resource lists, making check-in phone calls with patients, assist social and nurse navigator with needs
	Main Hospital:	Patient navigators assist in coordination of care services such as scheduling as well as prescription assistance. Additionally, the navigators connect patients to the following resources if needed:
E	Disease site-specific navigators assist patients	
	Satellite Sites:	
	General navigators cover all disease types	Case managers who assist with following up on the social determinants of health Integrative medicine Wellness program (exercise, yoga, and nutrition services) Financial assistance Transportation Counseling Rehabilitation services
	Main Hospital:	Services to address nonmedical barriers to care:
F	Nurse navigators provide disease-specific navigation	
	Satellite sites:	Education Care coordination, including appointment scheduling if needed Assistance with prescriptions, if needed Connection to the following resources: financial assistance, psychological/emotional support services, housing assistance, transportation assistance, food assistance
	Nurse navigators provide navigation regardless of cancer type	
	Main Hospital:	Navigators operate slightly differently depending on cancer type. However, generally, navigators coordinate care, provide emotional support, and connect patients to the additional resources listed below:
G	Nurse navigators meet with patients either before or at point of diagnosis Nurse navigators follow the patient throughout treatment progression and follow up on dynamic needs	Transportation assistance Hospitality house Financial navigation team Cancer wellness program (physical routine, exercise, yoga) Smoking cessation program Social worker counseling Support groups Dietician services Clinical trials CareNet volunteers (peer support program with patients who have previously gone through treatment) Pastoral care Palliative care Fertility preservation Genetic counseling

**Table 3.** Cancer Patient Navigation Configurations and Referral Patterns Among North Carolina Cancer Hospitals, 2023

Hospital	Diagnosis	Treatment
A	Pre-consult visit: patient is identified/contacted and barrier assessment is conducted prior to the patient’s first appointment. Barrier mitigation is initiated and a patient relationship is formed. Post-consult: Patients are referred to OPNs who are responsible for pre-treatment assessment and ongoing assessments, resourcing and support throughout treatment	OVNs follow-up during treatment duration through support visits while inpatient or in the treatment rooms and provide outreach calls as necessary.
B	Patients are contacted preferably before the first visit or after if needed. Barrier assessment is completed.	Patients are followed throughout their treatment.
C	Upon receiving a referral, patients are contacted preferably before the first visit or at the point a need/barrier is identified.	Patients are followed throughout their treatment.
D	Disease site-specific nurse navigators meet with patients at initial diagnosis and stay involved during treatment duration.	Social work navigators conduct a distress screening after the 1^st^ appointment and follow-up on resource needs throughout treatment duration. Additional distress screenings occur at radiation treatment start, and radiation treatment completion.
E	Nurse navigators connect with the patient preferably at their first oncology visit to establish care Nurse navigators complete barrier assessments alongside social workers	Nurse navigators follow up with patients in person frequently
F	Navigators focus heavily on the beginning of the patient’s journey to support them with education, resources and emotional support to expedite diagnosis, consultations, tests, procedures, treatment planning and initiation	Navigators are available after treatment begins if needed
G	For breast, lung, and GI patients, navigators may intervene at the point of biopsy. In other circumstances, navigators may reach out to the patient following diagnosis. Lastly, patients can selfrefer for these services if desired. During this first point of contact, navigators assess needs and barriers to care.	Navigators assist the patient throughout treatment and followup on needs. Navigators try to connect with patients in person as much as possible.

Footnote: Cancer hospital D reported final distress screening at their first survivorship visit at which point they were transitioned to a survivorship program external to patient navigation.

**Table 4.** Future Direction of Cancer Patient Navigation Programs Among North Carolina Cancer Hospitals, 2023

Hospital	Future Direction of Patient Navigation Program at Health System	Desired Future Direction of Patient Navigation in General
A	Cover all cancer diagnoses in all UNC cancer facilities in the state	Increase of patient navigation programs to provide comprehensive medical cancer care with a holistic, wrap around approach for patients and families while mitigating the non-medical barriers to care to promote optimal cancer outcomes.
B	Work to come up with new ways to triage patients based on need for navigation services	Patient navigation must work towards financial sustainability Would like payers to make these critical services reimbursable
C	Increase the number of navigators across all disease groups Employ improved data tracking and charting tools Work to more clearly understand the downstream income generated by navigators	An expansion of patient navigation in chronic disease and on a community level
D	Bolster of the lay navigator program Work to demonstrate the return on investment through data in order to display that this is valuable care Work to understand how to follow patients more closely Strengthen patient participation in support and survivorship programs	Want to see more programs involved in the academy of Oncology Nurse & Patient Navigators, AONN Standardization of acuity tools across intuitions
E	Increase number of patient navigators Include survivorship navigator within the program Add additional resources for patients Shift towards disease-specific navigators at satellite facilities as opposed to general navigators	Hope that navigation does not become a billed service so that this essential program may remain easily accessible to all patients
F	Update the standard work for patient navigators across the health system Bolster the forms and documentation in order to improve data collection Expand services to all patients	More research to acquire benchmark data about the number of patients each navigator should take on
G	Maintain or increase the number of navigators to adequately cover patients Expand services by establishing more multidisciplinary clinics	Support across health care for navigation Coverage for navigation services by payers without cost to the patient Greater accessibility to these essential programs for patients while also increasing the financial sustainability of these programs for health systems
